# Metabolic phenotype of male obesity-related secondary hypogonadism pre-replacement and post-replacement therapy with intra-muscular testosterone undecanoate therapy

**DOI:** 10.1007/s12020-017-1516-x

**Published:** 2018-02-02

**Authors:** Georgios K. Dimitriadis, Harpal S. Randeva, Saboor Aftab, Asad Ali, John G. Hattersley, Sarojini Pandey, Dimitris K. Grammatopoulos, Georgios Valsamakis, Georgios Mastorakos, T. Hugh Jones, Thomas M. Barber

**Affiliations:** 1 Clinical Sciences Research Laboratories, Warwick Medical School, University of Warwick, Clifford Bridge Road, Coventry, CV2 2DX UK; 2 Warwickshire Institute for the Study of Diabetes, Endocrinology and Metabolism (WISDEM) and the Human Metabolism Research Unit (HMRU), UHCW NHS Trust, Clifford Bridge Road, Coventry, CV2 2DX UK; 3 Division of Endocrinology and Investigative Medicine, Imperial College London, Hammersmith Campus, Du Cane Road, London, W12 0NN UK; 4 School of Engineering, University of Warwick, Coventry, CV4 7AL UK; 5 Pathology Labs, UHCW NHS Trust, Clifford Bridge Road, Coventry, CV2 2DX UK; 6 Unit of Endocrinology, Diabetes Mellitus and Metabolism, Aretaieio University Hospital, Athens Medical School, 11528 Athens, Greece; 7 Centre for Diabetes and Endocrinology, Barnsley District General Hospital, Gawber Road, Barnsley, S75 2EP UK; 8 Department of Oncology and Metabolism, Univerity of Sheffield, Sheffield, S10 2RX UK

**Keywords:** Male obesity-associated secondary hypogonadism, Testosterone replacement, Obesity, Secondary Hypogonadism, Testosterone, Type 2 Diabetes Mellitus

## Abstract

**Aim:**

To explore the metabolic phenotype of obesity-related secondary hypogonadism (SH) in men pre-replacement and post-replacement therapy with long-acting intramuscular (IM) testosterone undecanoate (TU).

**Methods:**

A prospective observational pilot study on metabolic effects of TU IM in male obesity-related SH (hypogonadal [HG] group, *n* = 13), including baseline comparisons with controls (eugonadal [EG] group, *n* = 15). Half the subjects (*n* = 7 in each group) had type 2 diabetes mellitus (T2D). Baseline metabolic assessment on Human Metabolism Research Unit: fasting blood samples; BodPod (body composition), and; whole-body indirect calorimetry. The HG group was treated with TU IM therapy for 6–29 months (mean 14.8-months [SD 8.7]), and assessment at the Human Metabolism Research Unit repeated. T-test comparisons were performed between baseline and follow-up data (HG group), and between baseline data (HG and EG groups). Data reported as mean (SD).

**Results:**

Overall, TU IM therapy resulted in a statistically significant improvement in HbA1C (9 mmol/mol, *P* = 0.03), with 52% improvement in HOMA%B. Improvement in glycaemic control was driven by the HG subgroup with T2D, with 18 mmol/mol [*P* = 0.02] improvement in HbA1C. Following TU IM therapy, there was a statistically significant reduction in fat mass (3.5 Kg, *P* = 0.03) and increase in lean body mass (2.9 kg, *P* = 0.03). Lipid profiles and energy expenditure were unchanged following TU IM therapy. Comparisons between baseline data for HG and EG groups were equivalent apart from differences in testosterone, SHBG and basal metabolic rate (BMR).

**Conclusion:**

In men with obesity-related SH (including a subgroup with T2D), TU IM therapy improved glycaemic control, beta cell function, and body composition.

## Introduction

Weight-gain in men, with resultant obesity is commonly associated with the development of obesity-related secondary hypogonadism (SH) [1, 2]. There are complex interlinks between the development of obesity-related SH and other metabolic dysfunctions including impaired glucose tolerance, type 2 diabetes mellitus (T2D), hypertension, dyslipidaemia, and obstructive sleep apnoea (OSA). Pathogenic factors are complex and multidirectional, and likely to include suppressive effects of oestradiol on the hypothalamo-pituitary gonadotrophic control of testicular testosterone production (and spermatogenesis) through increased aromatization of testosterone within visceral adipocytes [1]. Other mechanisms are likely to involve leptin, pro-inflammatory cytokines, and changes in body composition that result in the development of testosterone deficiency [1]. Obesity-related SH is a common condition and in one prospective study on 100 Spanish men with moderate-to-severe obesity, prevalence of obesity-related SH was reported as 45% [3]. In a further study from the Netherlands on 160 obese men, obesity-related SH (based on total testosterone levels) was present in 57.5% of the sample [4]. It should be noted, however, that levels of plasma testosterone are reduced spuriously by non-fasting status [5]. There is some controversy therefore regarding the true prevalence of obesity-related SH, and future studies in this field should avoid use of non-fasting measurements of plasma testosterone levels.

It is well-established that gonadotrophin-mediated reductions in plasma testosterone levels are observed in men with all forms of chronic disease or acute illness [6]. Furthermore, a general lack of awareness amongst healthcare professionals regarding male obesity-related SH as a clinical entity, and its appropriate screening and management remain contentious. It is likely that many cases of male obesity-related SH remain undiagnosed and untreated. Furthermore, the pathogenic mechanisms that underlie development of obesity-related SH in men, its association with metabolic dysfunction and the effects of testosterone replacement therapy (TRT) remain incompletely understood.

Improvement of insulin sensitivity through administration of TRT in hypogonadal men has attracted less controversy, with a number of published studies confirming such an effect [7]. In one study, TRT was shown to improve insulin sensitivity in subjects with T2D, demonstrated through improved glucose disposal rate during hyperinsulinaemic euglycaemic clamp [8]. A further study on androgel in men with partial androgen deficiency of aging showed reduction of visceral fat and improvement of insulin resistance [9]. These observations have been corroborated by data from animal studies that have also shown benefits of TRT on components of glucose uptake [10]. Furthermore, in vitro studies on the effects of testosterone in human subcutaneous adipocytes have shown increased expression of Glucose transporter type 4 (GLUT4) and Protein Kinase B (Akt), which in turn would be expected to facilitate enhanced glucose uptake and insulin sensitivity [8]. There are few published studies, however, that report on the effects of testosterone on beta cell insulin release in hypogonadal men. Of note, there were no changes in HOMA %B demonstrated with TRT in hypogonadal men in the TIMES 2 study [11].

The primary outcome of this study was change in HbA1C in obese hypogonadal men following testosterone undecanoate intramuscular (TU IM) therapy. Secondary outcomes following TU IM therapy in this group included changes in measures of insulin sensitivity and beta cell functioning (HOMA2 IR and HOMA %B, respectively), metabolic profile, body fat and lean mass, and fasting lipid profile. A further secondary outcome was a (baseline) metabolic comparison between obese hypogonadal and obese eugonadal men. The study reported is observational rather than a clinical trial. All administration of TU IM therapy was based on clinical need. To our knowledge, our study includes the first detailed assessment of metabolic rate through use of indirect calorimetry in a whole-body calorimeter (WBC) in men with obesity-related SH. This pilot study has two main components: (i) a longitudinal observational study on the metabolic effects of TU IM therapy in men with obesity-related SH, and; (ii) baseline comparisons of metabolic data from hypogonadal and eugonadal obese men. This report will be structured accordingly.

## Materials and methods

### Subjects

Adult, obese (BMI > 30 kg m^−2^) male subjects (*n* = 28) were recruited from the obesity clinic at the Warwickshire Institute for the Study of Diabetes, Endocrinology and Metabolism (WISDEM) Centre, University Hospitals Coventry and Warwickshire (UHCW) NHS Trust. The subjects were divided into two main groups. The first hypogonadal (“HG”) group (*n* = 13) included those with a confirmed diagnosis of obesity-related SH. This diagnosis was based on confirmed biochemical hypogonadism (9 am fasting serum testosterone <8 nmol/l on at least two separate days), with normal range levels of gonadotrophins LH and FSH. There were also clinical features of hypogonadism in all subjects within the HG group including either low libido and/or erectile dysfunction. In the HG group, other secondary causes of hypogonadism (such as prolactinoma, haemochromatosis, and congenital forms i.e., Kallman’s syndrome and compression effect from a pituitary mass) had been excluded. Furthermore, there were no other endocrine abnormalities. Within the HG group, seven subjects also had a confirmed diagnosis of T2D. Following baseline assessment on the Human Metabolism Reseach Unit (HMRU), all subjects in the HG group were commenced on TU 1000 mg injections (TU, Nebido®, Bayer AG, Germany) IM therapy for the duration of the study. TU IM was administered at 12-weekly intervals (with the second IM injection administered at 6-weeks following the first injection) as per prescribing guidelines.

The second eugonadal (“EG”) group (*n* = 15) included those with confirmed biochemical eugonadism with 9 am serum testosterone levels >10 nmol/l, normal-range gonadotrophins and absence of clinical features of hypogonadism. Within the EG group, a subgroup (*n* = 7) had a confirmed diagnosis of T2D. The duration of T2D and glycaemic therapies (for the subgroup of subjects with T2D in both the HG and EG groups) varied between 2 and 10 years. There were no exclusions regarding glycaemic therapies for those subjects with T2D, nor for statin or fibrate therapies. Only baseline assessment of the EG group on the HMRU was performed. Follow-up analyses were therefore restricted to the HG group following administration of TU IM therapy. The purpose of the EG assessment was to provide comparative data for the baseline metabolic assessment of the HG group.

Exclusion criteria included prior bariatric surgery, current/prior use of weight-loss therapies or any other treatment with androgenic or antiestrogenic properties. Use of prior TRT was a further exclusion criterion. None of the subjects had any existing or prior diagnosis of OSA or renal impairment at enrollment into the study. All clinical investigations were conducted in accordance with the guidelines in the Declaration of Helsinki. All subjects provided fully informed written consent prior to enrollment into the study, and a local Research Ethics Committee in the United Kingdom approved the study.

### Testosterone replacement therapy

Following baseline assessment on the HMRU, all hypogonadal subjects were initiated on TRT. For all subjects, the replacement used was TU IM therapy. The dosing schedule, as per prescribing guidelines, was as follows: Following an initial IM injection of TU 1 g, the second injection was administered at 6-weeks, and subsequent injections at 12-weekly intervals. TU IM injections were administered by the patient’s practice nurse based at their primary care setting. None of the subjects receiving TU IM therapy required venesection.

### Anthropometric, indirect calorimetric, energy balance, and sleep-based evaluations

All metabolic studies were conducted on the HMRU, described previously [12]. Subjects arrived at the HMRU for each of their assessments at 9 am in a fasting state (having fasted for 12-h prior to the study). Furthermore, subjects were advised to maintain a stable and isocaloric diet for 72 h before each of their visits (using a three day food questionnaire), whilst strenuous physical activity and caffeine ingestion were avoided for at least 24-h prior to each metabolic assessment on the HMRU. Following arrival at the HMRU and a fasting blood sample, each subject underwent anthropometric assessment of overall body fat and lean mass using a BodPod. The technique of body composition analysis from a BodPod is based on air displacement plethysmography (Cosmed Inc., USA) [13]. Body weight and height were measured.

Following anthropometric measurements, each subject entered a WBC, located at the HMRU at 1000 h, and was requested to lie on the bed for the first 30 min to allow for equilibration within the WBC. A macronutrient balanced breakfast meal was served at 1030 h, standard lunch at 1300 h, standard evening meal at 1800 h and standard snack at 2200 h. Subjects were requested to sleep between 2300 and 0730 h, and exited the WBC at 0800 h. The duration of each WBC study was therefore 22 h. Whilst inside the WBC, subjects were requested to avoid any physical activity. During each WBC study, minute-by-minute samples of air entering and leaving the WBC were measured accurately for levels of CO_2_ and O_2._ From these measurements of CO_2_ and O_2_, energy expenditure was calculated, providing a continous assessment of metabolic rate (Kcal/min) for the duration of each WBC study. Energy expenditure (EE) was calculated using Weir’s formula [14]: EE (Kcal) = [3.941 × O_2_ consumed (L)] + [1.106 × CO_2_ produced (L)]. Further details regarding the WBC have been described previously [12]. During each WBC assessment, relative humidity (57 ± 5%) and thermoneutral temperature (24 ± 0.5 °C) were tightly controlled. The timings and procedures for each study at the HMRU were identical for all metabolic assessments (including all baseline and all follow-up studies), to limit any potential for confounding effects. All subjects (including the HG and EG groups) underwent a baseline metabolic assessment at the HMRU. All those subjects in the HG group underwent a further follow-up metabolic assessment at the HMRU following TU IM replacement therapy for at least a 6-month period.

For each assessment at the HMRU, basal metabolic rate (BMR) was calculated from minute-by-minute EE during the 1-h nocturnal period (between 0500 h and 0559 h) when subjects were asleep. Thermic effect of food (TEF) was calculated from EE readings during the 4-hour period following the standard lunch (1300–1659 h) (EE–BMR). Finally, overall metabolic rate (21-h) whilst inside the WBC was calculated from the EE readings between 1030 and 0730 h for each WBC assessment. Sleep studies using an Alice PDX portable sleep machine (generously provided by Phillips Respironics) were performed on a subgroup of the subjects whilst sleeping inside the WBC, based on initial scoring on the Epworth test (sleep assessment conducted if a subject had a score of at least 10).

### Biochemical evaluation

Prior to each metabolic assessment at the HMRU, fasting blood samples were taken. Following immediate spinning of each sample in a centrifuge, serum was extracted and analyzed for lipid profile (total cholesterol, low-density lipoprotein (LDL) cholesterol, high-density lipoprotein (HDL) cholesterol, and triglycerides), HbA1C, glucose, insulin, testosterone, estradiol, prostate specific antigen (PSA), sex hormone binding globulin (SHBG), haemoglobin (Hb), and alanine transaminase (ALT). Serum insulin was measured with a chemiluminescent assay (DPC immulite 2500 machine). Glucose was measured using a hexokinase assay (ADVIA 2400 analyser). Serum testosterone and SHBG were measured using chemiluminescent assays (Immulite 2000 analyser). The normal reference range for testosterone in men using this assay was 8.6–29 nmol/l. Adiponectin and leptin were measured using a Sandwich ELISA (in vitro enzyme-linked immunosorbent) assay (Sigma). Intra-assay coefficient of variation for all assays used was <7%. Values for Homeostasis Model Assessment of Insulin Resistance (HOMA2 IR) and Homeostasis Model Assessment of Beta-cell function (HOMA %B) were calculated from paired measures of fasting plasma glucose and insulin levels for each subject at each of their visits at the HMRU, using the Oxford Diabetes Trials Unit HOMA2 calculator (https://www.dtu.ox.ac.uk/homacalculator/). Subjects on insulin therapy were excluded from HOMA analyses. Given the importance of considering levels of SHBG when assessing male androgenicity, we also calculated free (bioavailable) testosterone levels using the formula, free androgen index (FAI) = 100 × (total testosterone/SHBG), with units of testosterone and SHBG in nmol/l.

### Statistical analyses and power calculation

Statistical analyses were conducted in SPSS (version 22 Windows; SPSS Inc., Chicago, IL). Data for most analytes (including testosterone) were approximately normally-distributed. Paired two-tailed *t*-tests were used for comparisons between baseline and follow-up data (following treatment with TU IM replacement therapy) for each of the subjects in the HG group. For these analyses, each subject acted as their own control thereby limiting confounding. Independent sample *t*-tests were used for comparisons between baseline data from the HG and EG groups. We had >80% power to detect a between-group difference in HbA1C exceeding 100% of a standard deviation (SD) (alpha = 0.05). A *P*-value <0.05 was considered significant. Data are shown as mean and standard deviation (SD) unless otherwise stated.

## Results

### Longitudinal observation of the metabolic effects of TU IM therapy: paired-sample *t*-test comparisons between baseline and follow-up data in the HG group

In the hypogonadal (HG) group (*n* = 13), subjects were treated with TU IM therapy (TRT) for at least 6-months between baseline and follow-up metabolic assessments at the HMRU (mean [SD] duration of TU IM therapy 14.8 months [8.7]). The maximum duration of TU IM therapy between metabolic assessments was 29-months. Given the dosing schedule for TU IM therapy, all HG subjects had received at least four injections of TU at their follow-up study, with a mean number of six injections. Follow-up metabolic assessments for HG subjects were executed between 10 and 12-weeks following the last TU IM injection.

As expected, there was a significant improvement in serum trough testosterone levels between baseline and follow-up assessments (mean serum trough testosterone [SD] 6.7 nmol/l [5.9] vs. 15.2 nmol/l [6.4], *P* = 0.046; for baseline and follow-up assessments at the HMRU, respectively). There was also a significant improvement in FAI following TU IM therapy (mean FAI [SD] 28.3 [12.7] vs. 60.8 [35.5], *P* = 0.01; for baseline and follow-up assessments at the HMRU, respectively). TRT resulted in statistically significant improvements in anthropometric measures of body composition, with (mean) reduction in fat mass of 3.5 kg (*P* = 0.03) and (mean) increase in lean body mass of 2.9 kg (*P* = 0.03). There was also a 2.8% (*P* = 0.03) reduction in body fat percentage following TRT (data shown in Table 1).

**Table 1:** Comparison of anthropometric, biochemical and metabolic data between assessments performed at baseline and following treatment with TU IM testosterone replacement therapy (paired sample *t*-tests; bold p-values indicate statistical significance)

Phenotype	Baseline HG group mean (SD) (*n* = 13)	Follow-up HG group mean (SD) (*n* = 13)	Paired-sample *t*-test comparison
BMI (kg m^−2^)	38.5 (6.0)	38.0 (5.5)	0.18
Age (years)	49.4 (8.8)	N/A	N/A
Body weight (kg)	120.9 (18.6)	119.5 (16.3)	0.26
Fat mass (kg)	51.2 (15.8)	47.7 (16.1)	**0.033**
Lean mass (kg)	67.9 (7.0)	70.8 (6.6)	**0.028**
Body fat (%)	42.2 (7.1)	39.4 (8.8)	**0.030**
BMR (Kcal 5–6 am)	92.6 (14.7)	97.0 (13.4)	0.24
Metabolic rate 21 h (Kcal)	2316 (353)	2321 (328)	0.90
Thermic effect of food (Kcal)	495 (80)	498 (73)	0.78
Hb (g/dl)	15.2 (1.1)	15.7 (1.1)	0.16
ALT (U/l)	36 (15)	29 (13)	0.11
HbA1C (mmol/mol)	56 (16)	47 (7)	**0.028**
Fasting glucose (mmol/l)	9.3 (3.3)	6.8 (1.9)	0.10
Fasting insulin (pmol/l)	270 (188)	240 (133)	0.51
HOMA2 IR	5.5 (3.8)	4.5 (2.4)	0.32
HOMA %B	108 (46)	160 (78)	**0.05**
Total cholesterol (mmol/l)	4.1 (1.0)	4.0 (0.8)	0.74
HDL cholesterol (mmol/l)	1.1 (0.1)	1.1 (0.2)	0.19
LDL cholesterol (mmol/l)	2.4 (1.0)	2.2 (0.8)	0.45
Triglycerides (mmol/l)	1.7 (0.7)	1.8 (0.9)	0.61
Adiponectin (pg/ml)	1520 (461)	1489 (320)	0.87
Leptin (pg/ml)	164 (99)	144 (99)	0.31
Estradiol (pmol/l)	97 (54)	194 (75)	**0.042**
PSA (ng/ml)	0.8 (0.5)	1.1 (0.7)	**0.022**
Testosterone (nmol/l)	6.7 (5.9)	15.2 (6.4)	**0.046**
SHBG (nmol/l)	21.3 (6.9)	25.0 (7.5)	**0.003**
Haematocrit (%)	45.5 (3.4)	47.2 (3.3)	0.19
Haemoglobin (g/dl)	14.8 (1.3)	15.3 (1.2)	0.16
Free androgen index	28.3 (12.7)	60.8 (35.5)	**0.01**

Most of the HG group had a confirmed diagnosis of T2D (*n* = 7), with the remainder euglycaemic (*n* = 6). There was a statistically significant improvement in glycaemic control amongst the whole HG group following TRT, with (mean) improvement in HbA1C of 9 mmol/mol (*P* = 0.03). Although there was a trend for HOMA IR reduction with TRT therapy (HOMA IR at baseline versus follow-up, 5.5 vs. 4.5, respectively, *P* = 0.32), this did not reach statistical significance. There was however, a significant improvement in beta-cell function following TRT (mean HOMA %B improved by 52%), despite only non-significant reductions in fasting glucose and insulin levels (Table 1). Comparisons between baseline and follow-up HMRU assessments of overall metabolic rate and TEF showed no significant changes with TRT. Although there was a numerical increase in BMR following TRT, this also failed to reach significance (Table 1). There were no changes in lipid parameters following TRT. There was a significant (mean) small increase in PSA by 0.3 following TRT, although PSA remained well within the normal range in all subjects. Following TRT, serum estradiol level doubled (*P* = 0.04) and there was a significant increase in SHBG by 3.7 nmol/l (*P* = 0.003). (Data are shown in Table 1).

Subgroup analyses revealed that the improvements in HbA1C with TRT for those subjects in the HG group were confined to the subgroup with an established diagnosis of T2D (*n* = 7). Within this subgroup, baseline and follow-up (mean) HbA1C levels were 70 [SD 9.2] vs. 52 mmol/mol [SD 4.0], *P* = 0.023, respectively. There was no significant change in HbA1C for the euglycaemic HG subgroup (*n* = 6), with baseline and follow-up (mean) HbA1C levels 42.6 [SD 3.9] vs. 41.2 mmol/mol [SD 4.3], *P* = NS respectively.

### Baseline (independent *t*-test) comparisons of metabolic data between the HG and EG groups

Data from baseline measurements in the HG group were compared with those from a control eugonadal (EG) group (*n* = 15). Within the EG group, a subgroup (*n* = 7) had a confirmed diagnosis of T2D, and the remainder (*n* = 8) had confirmed euglycaemia, and no present or past history of DM. As per definition, there was a statistically significant difference in (mean) baseline serum testosterone levels between the HG and EG groups (baseline serum testosterone 6.7 nmol/l [SD 5.9] vs. 14.2 nmol/l [SD 5.0], *P* = 0.036 respectively). There was also a significant difference in baseline serum SHBG levels between the groups (baseline serum SHBG 21.3 nmol/l [SD 6.9] vs. 37.8 nmol/l [SD 19.6], *P* = 0.010 for HG and EG groups, respectively). Interestingly, despite there being similar lean mass at baseline between the HG and EG groups, the HG group had a statistically greater BMR at baseline than the EG group (92.6 vs. 79.9 Kcal, respectively, *P* = 0.040, Table 2). All other baseline comparisons between the HG and EG groups were statistically equivalent (data shown in Table 2).

**Table 2:** Comparison of anthropometric, biochemical, and metabolic data between assessments performed at baseline for hypogonadal (HG) and eugonadal (EG) groups (independent sample *t*-tests; bold p-values indicate statistical significance)

Phenotype	Baseline HG group mean (SD) (*n* = 13)	Baseline EG group mean (SD) (*n* = 15)	Independent-sample *t*-test comparison
BMI (Kg m^−2^)	38.5 (6.0)	36.6 (5.5)	0.47
Age (years)	49.4 (8.8)	50.4 (12.1)	0.81
Body weight (kg)	120.9 (18.6)	110.5 (18.2)	0.27
Fat mass (kg)	51.2 (15.8)	43.9 (17.6)	0.34
Lean mass (kg)	67.9 (7.0)	65.0 (6.1)	0.33
Body fat (%)	42.2 (7.1)	39.1 (9.7)	0.46
BMR (Kcal 5–6 am)	92.6 (14.7)	79.9 (10.8)	**0.040**
Metabolic rate 21 h (Kcal)	2316 (353)	2065 (287)	0.12
Thermic effect of food (Kcal)	495 (80)	441 (65)	0.17
ALT (U/l)	36 (15)	28 (25)	0.30
HbA1C (mmol/mol)	56 (16)	49.2 (21.5)	0.33
Fasting glucose (mmol/l)	9.3 (3.3)	6.7 (1.0)	0.11
Fasting Insulin (pmol/l)	270 (188)	141 (74)	0.09
HOMA2 IR	5.5 (3.8)	2.7 (1.4)	0.07
HOMA %B	108 (46)	108 (49)	0.65
Total cholesterol (mmol/l)	4.1 (1.0)	4.2 (0.8)	0.99
HDL cholesterol (mmol/l)	1.1 (0.1)	1.2 (0.4)	0.72
LDL cholesterol (mmol/l)	2.4 (1.0)	2.2 (0.8)	0.64
Triglycerides (mmol/l)	1.7 (0.7)	1.5 (0.6)	0.77
Adiponectin (pg/ml)	1520 (461)	2833 (2773)	0.28
Leptin (pg/ml)	164 (99)	99 (72)	0.17
Estradiol (pmol/l)	97 (54)	115 (48)	0.92
PSA (ng/ml)	0.8 (0.5)	2.3 (4.9)	0.34
Testosterone (nmol/l)	6.7 (5.9)	14.2 (5.0)	**0.036**
Luteinizing hormone LH (IU/l)	4.6 (2.6)	6.4 (3.5)	0.18
Follicle stimulating hormone FSH (IU/l)	5.6 (4.7)	7.9 (5.3)	0.28
SHBG (nmol/l)	21.3 (6.9)	37.8 (19.6)	**0.010**
Haematocrit (%)	45.5 (3.4)	44.9 (2.1)	0.79
Haemoglobin (g/dl)	14.8 (1.3)	15.0 (1.0)	0.77
Free androgen index	28.3 (12.7)	37.9 (15.6)	0.14

### Sleep data

Overall, polysomnography (using a portable Alice PDX sleep machine) was assessed in 13 subjects at baseline on the basis of an Epworth score of at least 10 (including those from the HG [*n* = 6]) and EG [*n* = 7] groups). Of these, there were 9 (32% of the recruited subjects overall) from the HG (*n* = 5) and EG (*n* = 4) groups who had newly-diagnosed and treatable OSA (defined by an apnoea-hypopnoea index [AHI] > 15/h). Of these, 4 (14% of the recruited subjects overall; two from each group) had severe OSA (AHI > 30/hour). Sleep studies were not repeated following TRT. The nine subjects who were diagnosed with treatable OSA were treated with continuous positive airways pressure therapy. Of these nine subjects, five also had T2D (three within the HG group and two within the EG group).

### Changes to glycaemic therapies in the HG subgroup with T2D during treatment with TRT

In the HG subgroup with T2D (*n* = 7), baseline glycaemic management was as follows: lifestyle (*n* = 1); metformin monotherapy (*n* = 2); dual therapy with metformin and liraglutide (*n* = 1); dual therapy with meformin and sitagliptin (*n* = 1); quadruple therapy with metformin, pioglitazone, gliclazide and liraglutide (*n* = 1), and; triple therapy with metformin, liraglutide, lantus and novorapid (*n* = 1). During TRT and prior to the follow-up visits, glycaemic therapies remained unchanged in four subjects. In one subject, dual therapy with metformin and liraglutide was intensified by the addition of levemir (12 units od). In one subject, dual therapy with metformin and sitagliptin had been changed to metformin and liraglutide. In one subject, quadruple therapy with metformin, pioglitazone, gliclazide and liraglutide was changed to metformin, pioglitazone, lantus (20 units od) and canagliflozin. Details of glycaemic therapies for the HG subgroup with T2D are shown in Table 3.

**Table 3:** Comparison of glycaemic therapies at baseline and follow-up assessments on the Human Metabolism Research Unit for the HG subgroup with T2D

Subject number	Glycaemic therapies at baseline	Glycaemic therapies at follow-up assessment
1	Lifestyle	Unchanged
2	Metformin	Unchanged
3	Metformin	Unchanged
4	Metformin	Addition of Levemir (12 units)
	Liraglutide	
5	Metformin	Sitagliptin changed to Liraglutide
	Sitagliptin	
6	Metformin	Gliclazide and Liraglutide changed to Lantus (20 units od) and Canagliflozin
	Pioglitazone	
	Gliclazide	
	Liraglutide	
7	Metformin	Unchanged
	Liraglutide	
	Lantus	
	Novorapid	

## Discussion

In this well-phenotyped study on the metabolic effects of TU IM therapy in obese men with secondary hypogonadism, we demonstrate significant improvements in glycaemic control (likely mediated at least in part through improved beta-cell function [HOMA %B]) and favorable changes in body composition, consistent with several other studies reported with TRT [11, 15–17]. With TRT, there was also a statistically significant improvement in SHBG (known to correlate with insulin sensitivity and BMI in obese men) [18, 19]. The observed significant increase in serum estradiol levels following TU IM therapy in the HG group likely reflects aromatization of exogenously-administered testosterone within adipocytes. The lack of significant changes in metabolic rate and fasting lipid profiles with TU IM therapy may be explained by lack of power, the relatively small changes in body composition following TU IM therapy, and possible changes in dietary and lifestyle factors (such as propensity for exercise) amongst hypogonadal subjects during TU IM therapy. The significant difference in baseline BMR between the HG and EG groups is harder to explain, given the similarity of lean mass between the two groups. Given the apparent lack of effect of TRT on metabolic rate in the HG group, it is difficult to attribute the difference in baseline BMR between the HG and EG groups to differences in testosterone. It remains possible that differences in other features between the two groups that were not measured (such as physical fitness for example) may, at least partly explain this difference, although this is purely speculative.

In one of the largest studies on TRT in hypogonadal men published to date (TIMES2 study), Jones and Colleagues reported a 16.4% improvement in HOMA IR following administration of transdermal 2% testosterone gel over 12-months in 220 hypogonadal men with T2D and/or metabolic syndrome [11]. In the subgroup with T2D, there was an improvement in HbA1C and in total and LDL cholesterol [11]. In another study reported by Francomano and colleagues, the metabolic effects of testosterone injections were compared with dietary change and physical activity in severely obese men with hypogonadism (*n* = 24) [20]. Following 1 year of testosterone injections combined with improved lifestyle, there were significant improvements in HOMA-measures of insulin sensitivity, total cholesterol, and inflammatory parameters [20].

The longer-term metabolic effects of TU IM therapy (administered for up to 60-months) were reported by Traish and Colleagues who performed an observational study on 255 (mainly elderly) men with sub-normal testosterone levels [15]. Overall, there were improvements in parameters of metabolic syndrome including lipid profile and blood pressure. In the subgroup with T2D (*n* = 81), there were also significant improvements in HbA1C, consistent with our own data. In a further study by Haider and colleagues on obese men with hypogonadism (*n* = 181; *n* = 72 with T2D), long-acting intramuscular testosterone undecanoate administered for up to 5-years resulted in significant improvements in waist circumference, BMI, lipid profile and glycaemic control [16]. A long-term study by Saad and Colleagues on 411 obese and hypogonadal men who received TU IM therapy for a maximum duration of 8-years, and a mean follow-up of 6-years, demonstrated sustained weight loss and reduced waist circumference [17]. Finally, in a observational, prospective and cumulative registry of 656 hypogonadal men, including 360 hypogonadal men who had received TU IM therapy for up to 10 years (the remainder who had opted against TRT), significantly improved mortality (66–92%) related to CV disease was shown in the treatment group [21].

Grillo and colleagues reported that testosterone resulted in a rapid and dose-dependent increase of insulin release from isolated pancreatic islets of Langerhans, in the presence of non-stimulatory glucose concentrations [22]. A further study on male mice lacking the androgen receptor in their beta cells, demonstrated reduced glucose-stimulated insulin secretion (GSIS) [23]. Dihydrotestosterone (DHT)-induced enhancement of GSIS in cultured human islets is abolished through treatment with an androgen receptor antagonist [23]. Interestingly, in both mouse and human islets, activation of the glucagon-like peptide-1 receptor appeared to mediate the insulinotropic effects of DHT [23]. A further rodent-based study showed that gene expression within islets (including genes implicated in insulin secretion and inflammation) is altered with androgen receptor knock-out [24]. Observations of deterioration of glycaemic control with androgen deprivation therapies for prostate cancer, combined with HOMA %B analyses [25, 26] are consistent with the in vitro data outlined above [23]. Data from other studies suggest that gonadal steroids, including testosterone may influence insulin secretion in a sex-specific manner [27].

Our own study demonstrated an improvement in HOMA %B in those hypogonadal men with T2D following TU therapy. We hypothesize that improvement in HOMA %B in this subgroup is mediated, at least in part through direct effects of testosterone on beta-cell insulin release, consistent with observations by Grillo [22] and Navarro [23] as outlined above. Further studies are required to explore direct effects of testosterone on beta-cell function, and the potential therapeutic role of such a mechanism on improved glycaemic control in hypogonadal men with T2D.

An important limitation of our study is that the numbers included in each of the HG and EG groups is small. This limits the power of our data, including for example the lipid profiles, which need to be interpreted with care. Furthermore, given the well-established association between BMR and lean body mass [28], it is possible that significant changes in BMR would have been discerned with a larger cohort. A further limitation is the relatively short duration of TU IM therapy between baseline and follow-up HMRU studies (at least 6-months). In three of the HG subgroup with T2D, glycaemic therapies were changed (on the basis of clinical need) during therapy with TU IM therapy and prior to their follow-up visit. The glycaemic effects of these therapy changes have potential to confound the glycaemic effects of TU IM therapy in this subgroup. Moreover, differences in the duration of glycaemic therapies in subjects with T2D in the HG group prior to their enrollment into the study may have also influenced response to TU IM therapy. Additionally, sleep studies were not repeated following TRT. It has been reported previously that TRT is associated with improved fatigue, energy, and vigor [29]. Future studies should assess the role of sleep improvement with TRT as a potential mediator of this association. Our study was also limited by the lack of a placebo arm and follow-up data for the EG group, which has the potential to confound follow-up metabolic data. However, although effects on health-related behavior in the HG group are possible through inclusion in this study, subjects were only required to attend for one baseline and one follow-up metabolic assessment, and otherwise received standard clinical care and follow-up throughout the duration of the study.

In summary, we demonstrate, in a prospectively-designed and highly-phenotyped study, evidence that TU IM therapy improves glycaemic control and body composition in men with obesity-related SH (including a subgroup with T2D), the former mediated at least in part through improved beta-cell function. The similar metabolic profiles between obese HG and EG men should lower the threshold for screening for hypogonadism in obese men. Longer-term placebo-controlled randomized studies on the metabolic and cardiovascular effects of TU IM therapy in obese HG patients (including those with T2D) are required, to gain mechanistic insight, and to further inform clinicians of the utility, efficacy, and potential risks of such a treatment option.

## Abbreviations

AHI: Apnoea-hypopnoea index
ALT: Alanine transaminase
BMI: Body Mass Index
BMR: Basal metabolic rate
DHT: Dihydrotestosterone
DM: Diabetes mellitus
EE: Energy expenditure
EG: Eugonadal
FAI: Free androgen index
FSH: Follicle stimulating hormone
GSIS: Glucose-stimulated insulin secretion
Hb: Haemoglobin
Hct: Haematocrit
HG: Hypogonadal
HMRU: Human Metabolism Research Unit
HOMA %B: Homeostasis model assessment of beta-cell function
HOMA2 IR: Homeostasis model assessment of insulin resistance
IM: Intramuscular
LH: Luteinizing hormone
NS: Non-significant
OSA: Obstructive sleep apnoea
PSA: Prostate specific antigen
SD: Standard deviation
SH: Secondary hypogonadism
SHBG: Sex hormone binding globulin
TEF: Thermic effect of food
T2D: Type 2 diabetes mellitus
TRT: Testosterone replacement therapy
TU: Testosterone undecanoate
UHCW: University Hospitals Coventry and Warwickshire
WBC: Whole-body calorimeter
WISDEM: Warwickshire Institute for the Study of Diabetes, Endocrinology and Metabolism
